# What Happens to the Health of Elderly Parents When Adult Child Migration Splits Households? Evidence from Rural China

**DOI:** 10.3390/ijerph17051609

**Published:** 2020-03-02

**Authors:** Tianxiang Li, Beibei Wu, Fujin Yi, Bin Wang, Tomas Baležentis

**Affiliations:** 1 College of Economics & Management, Nanjing Agricultural University, Nanjing 210095, China; garylee0428@gmail.com (T.L.); fujinyi@njau.edu.cn (F.Y.); 2 Anhui Institute of Economics, Hefei 230000, China; wbjobs@126.com; 3 Lithuanian Institute of Agrarian Economics, Kudirkos 18-2, LT-03105 Vilnius, Lithuania; tomas@laei.lt

**Keywords:** adult child migration, parental health, inter-generational support, instrumental variables

## Abstract

There is little understanding about the effects of adult child migration on the health of elderly parents left behind in the light of economic contribution and time allocation (farm work and emotional cohesion). Using the pooled data from three latest issues of China Health and Retirement Longitudinal Study (CHARLS) in the rural areas, this study assesses the impact of child migration on parents’ health by employing instrumental variable approach to deal with the endogeneity problem. Overall, the evidence suggests that adult child migration impairs parental health as indicated by lower self-reported health (SRH), body mass index (BMI), physical activity of daily living (PADL), and higher depression score. Moreover, parents who are female, poorly-educated, and living with one adult child at least are the most vulnerable groups in terms of poor health outcomes. The negative impact of farming burden on the health of parents left behind outweighs the positive impact of economic support and emotional cohesion. Thus, child migration exerts a significantly negative impact on parental health. Establishing medical and social security systems for the elderly is important to complement the traditional family support in rural China.

## 1. Introduction

China has become an aging society since the beginning of the new century [1,2]. According to the 2019 Chinese census, the number of people aged 60 years or above in China has reached 249 million by the end of 2018, accounting for 17.9% of the total population in the country. Population aging has posed potential challenges on the social security systems in China, especially in rural areas [3]. Due to limited coverage and insufficient benefits provided by public pension and health insurance systems [4], adult children remain the primary source of financial and instrumental support for their elderly parents [1]. Although the new cooperative medical scheme in rural China has substantially improved rural residents’ use of healthcare services, it has not reduced out-of-pocket expenses for patients [5]. Therefore, the traditional home-based care for the elderly has been challenged, especially when their adult children are migrant [6]. The massive migration of younger population from rural to urban areas has threatened to erode the foundation of traditional family support relationships [7,8,9]. Therefore, investigating how adult child migration affects the health of left-behind parents comprises an important research direction.

A number of studies have dealt with the relationship between adult child migration and their parental health (e.g., [10,11,12,13]). The literature provides mixed results for the impacts of child migration on parental health (e.g., [2,14,15]). Intuition suggests that adult child migration is detrimental to health of the left-behind parents (e.g., [4,13,16]). For example, Evandrou et al. [13] indicated that the odds ratio of reporting a disease was higher among older parents with at least one adult child living in another county. Adult child migration indeed increases economic support for left-behind parents, but it forces parents to undertake more intensive farming tasks. Child migration, therefore, may induce the absence of adult children at home, less attention and negative effect on parental health.

Other empirical studies find that the elderly with migrant children may experience better health status [2,8]. Ma and Zhou [14] indicated that the positive impact of adult children migration induced by increased money transfer to the elderly may offset the negative impact arising from decreasing physical care. Yi et al. [2] also found that the loss of labor power due to migration has a significantly negative effect on parental health, but remittances from migrants can compensate for the adverse effect. Overall, left-behind elderly parents may benefit from migrant children both physically and mentally.

Thus, the empirical evidence on the impact of adult child migration on left-behind parents’ health remains vague. A possible reason may be that the mechanism of time allocation is not clear, although previous literature has generally considered that children migration can improve parental health due to remittances. To the best of our knowledge, few studies have defined and identified the time effect on parental health. For example, Yi et al. [2] only discussed the impact of time allocation on the health of left-behind parents along with the negative effect caused by labor loss. In our study, we consider the twofold time effect—increased farm work driven by child migration and emotional cohesion—for the first time. In this setting, the effect of time allocation on the health of left-behind parents may be positive or negative. This can be related to the modern telecommunication technologies that are helpful to alleviate the elderly’s loneliness, dispel their negative emotions, and improve their physical and mental health [17]. The main problem is to distinguish its total net effects (i.e., income transfer, increased farm work, and emotional cohesion) on the health of the left-behind parents.

The issue of endogeneity has not been fully addressed in some of the previous studies, which may be led to biased estimation. One potential obstacle of estimating the causal effect of child migration on the parental health is reverse causality. Giles and Mu [11] found that the parental health conditions affect the migration decision of their adult children. The methodological challenges have given rise to a series of innovative approaches, including counterfactual framework [18], quasi-randomized experiments [19], and instrumental variables. A number of studies have attempted to find instrumental variables for migration (e.g., [2,13,20,21,22,23]), such as migration rates (e.g., [22]), local industry (e.g., [2]), and ecological variables (e.g., [23]). Thus, the instrumental variable estimation is the most popular approach to identify selection into migration.

Few studies have examined the spouse’s health condition and the living arrangement [1], which are also important to gauge the relationship between migrant children and parental health. Without accounting for the aforementioned variables, the estimates may be biased.

As stated above, the transmission mechanism of adult child migration on parental health is missing or ambiguous in the earlier literature. Much of the previous research analyzes the impact of child migration on parental health from the perspective of financial support, but either ignores the time effect of adult child migration or simply argues that the time effect is negative [2]. In fact, the time effect is very complex due to the development of modern telecommunication technologies, which may to a large extent alleviate the negative impact of child migration on parents’ emotional solace [17]. Thus, it cannot be simply concluded that child migration exerts a negative impact on parental health. As stated above, the objective of this study is to investigate the impact of child migration on parental health in rural China, and gauge the effects of the three types of inter-generational support: income transfer, farm work, and emotional cohesion.

The remainder of this study proceeds as follows: Section 2 outlines the theoretical model, and discusses the choice of instrumental variables to resolve the reverse causality. Section 3 describes the data used in this study. The econometric model is discussed in Section 4. Section 5 presents the empirical findings, and Section 6 concludes with policy implications.

## 2. Theoretical Framework

The issues of public health, economics and migration are often intertwisted [24,25,26]. Under China’s rapid economic growth and urbanization, the traditional home-based elderly care in its rural area has been transforming because of adult child migration, among other factors. It has been reported that 88.7% of the elderly need assistance in their daily activities from family members [4], however, massive rural-to-urban migration in China has profoundly altered the living style of left-behind elderly [8]. Given the long distance to migrant children, their left-behind parents have to take care of themselves. There are two theoretical premises that may help to gauge the impact of child migration on parental health outcomes, including the effects of economic contribution and time allocation.

First, economic contribution is the core element of the elderly-care system, and it exerts a direct impact on life quality and health of left-behind parents. Migrant children often earn more in other regions, so they can offer better financial support to their parents, which might help to raise parents’ living quality and improve their access to better medical services [15]. Thus, in this sense, adult child migration might increase parents’ satisfaction with life and improve their health conditions. 

Second, time allocation from adult children could also impact elderly health. Migration makes children unable to provide daily care for left-behind parents, and the parents have to carry out more work on farm and housework. This may pose an adverse impact on their physical and mental health. Additionally, adult children’s spiritual consolation is also needed for their parents [27]. Several studies have found that modern telecommunication technologies are helpful to alleviate parents’ loneliness driven by child migration, dispel their negative emotions, and improve their physical and mental health [17,28].

**Figure 1:**
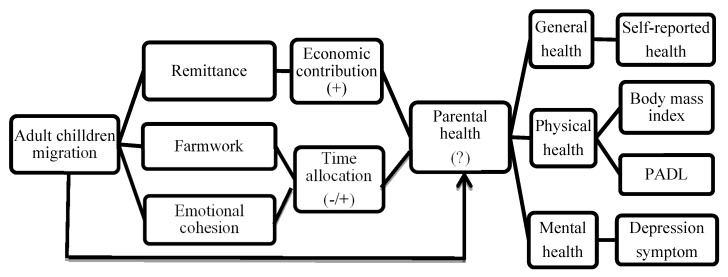
Conceptual framework for the impact of the child migration on parental health in rural areas. Notes: (1) A minus (resp. plus) sign indicates a negative (resp. positive) impact on the parental health; (2) PADL stands for physical activity of daily living.

As stated above, child migration has both positive and negative effects on parental health. The net effect of child migration on parental health depends on whether the positive effect (i.e., economic support and emotional cohesion) can compensate for the negative effect from the loss of family labor (i.e., increased farm work). This study investigates these two opposite effects through the above-mentioned channels of adult child migration on the health of left-behind elderly for the first time. Figure 1 presents the conceptual framework in the detailed manner.

## 3. Data

### 3.1. Data Source

The data used in this study come from the China Health and Retirement Longitudinal Study (CHARLS) conducted by the National School of Development at Peking University. Three waves of CHARLS have been conducted since 2011 on approximately 4400 households with a total of 16,000 individuals from 150 counties across 28 provinces in China. The survey covers areas which are diverse in geographic location, economic development, and health indicators. Counties in all provinces are stratified by income, and a multistage, randomized clustering process is then adopted. During the survey, persons aged 45 or older are selected and surveyed together with their spouses face-to-face by trained interviewers using structured questionnaires. The subjects covered in the survey include health status and functioning, health care and insurance, housing, assets, pensions, employment and retirement. A more detailed description of this survey is provided by Zhao et al. [29]. Our study focuses on the parental health conditions in rural China with migrant children, pooled data of three waves from CHARLS (2011, 2013, and 2015). The sample was narrowed to the adults who have at least one child living in rural areas. The final sample of pooled data contains 28,226 rural elderly, including 9660, 9337, and 9229 respondents in 2011, 2013, and 2015, respectively.

### 3.2. Variables

The variables used in this study are discussed in this section. The associated descriptive statistics are given in Table 1.

#### 3.2.1. Child Migration and Parental Health

Measurement of migration and health outcome is crucial for our study. The respondents were asked where the children normally lived at that time with seven response options. Having migrant children was assumed when there were children living in another area in the same province, or in another province [13]. Therefore, an elderly person having at least one migrant adult child is considered as a left-behind parent.

One of the main advantages of the CHARLS data for analyzing the health status of parents is that they provide multiple individual-level health indicators, including self-reported health (SRH), physical activity of daily living scale (PADL), body mass index (BMI), and depression score (CES-D). These variables are also widely used in other studies on the impact of child migration on elderly health [30]. A more detailed description of health indicators is given in Appendix A.

#### 3.2.2. Mediating Variables: Inter-generational Support

To reveal the potential transmission mechanism, this study explores the effect of child migration on both time allocation of parents and remittances parents received from migrant children. Two dimensions are used to investigate the impacts of child migration on time allocation: (1) daily care; and (2) emotional cohesion. First, with labor losses due to adult child migration, the elderly might have to spend more time and take higher responsibility in agricultural activity [31]. Thus, increased farming time is considered as a loss in daily care.

Second, emotional cohesion is defined as the aggregate frequency of emotional care that migrants provide for their parents left behind [17,32]. The respondents were asked the question “how often do you have contact with the children either by phone, text message, mail, or email, when you didn’t live with your children?” with higher scores indicating more frequent contacts. Indeed, frequent contacts are expected to improve parental health and well-being.

#### 3.2.3. Instrumental Variables

To address the possible reverse causality, we instrument the adult child migration with the migration rate and the number of factories in their own villages. The underlying reason is that the migration rate of co-villagers increases one’s propensity to migrate as co-villagers help each other in reducing the migration cost and assisting in job search at the destination [33]. More employment opportunities in local villages will dampen migration incentives [2]. Additionally, the two instrumental variables should have no direct effects on parental health conditions.

### 3.3. Descriptive Statistics

Table 1 provides the mean values of the variables used in the empirical models. A simple comparison of means for SRH, BMI, PADL and CES-D between parents with migrant children and those without indicates that parental health is negatively associated with child migration (Table 1). The cohorts with migrant children are more likely to report lower SRH (2.46 vs. 2.52), lower BMI (23.19 vs. 23.38), lower PADL (6.23 vs. 6.25), and high CES-D score (18.83 vs. 18.38). Thus, we now turn to focusing on the question how parental health responds to child migration.

## 4. Estimation Strategy

### 4.1. Econometric Model

Recognizing that decision on migration is probably not randomly assigned and migrants do self-select, it is generally acknowledged that migration is likely to be correlated with the same factors that influence outcomes for family members left behind [34]. Thus, it becomes difficult to determine whether migration is causing the outcome of interest or whether it is impacted by some other lurking variable that is correlated with both migration and the outcome of interest [4,11].

One potential obstacle to establishing the causal effect of children migration on the parental health is reverse causality. To address this issue, we adopt a two-stage instrumental variable (IV) approach. The IV estimation includes an outcome equation and a participation equation where the participation is binary and the outcome is linear. The outcome equation describing parental health can be expressed as:

(1) $${Health}_{i}=\beta_{0}+\beta_{1}X_{H}+\beta_{2}{Migration}_{i}+\mu_{H}$$

where *Health_i_* represents different health indicators for individual i defined in Table 1; *X_H_* is a set of observable covariates including household and individual characteristics (respondent’s age, gender, marital status, education, working status, chronic diseases etc.); *Migration_i_* represents adult child’s migration decision. The decision to migrate is defined as:

(2) $${Migration}_{i}=\alpha_{0}+\alpha_{1}X_{M}+\alpha_{2}IV_{M}+\mu_{M}$$

where ${Migration}_{i}$ = 1 denotes decision to migrate and ${Migration}_{i}$ = 0 is observed otherwise; $X_{M}$ is a vector of other control variables as discussed above, including the characteristics of household and children, *IV_M_* is a vector of instrument variables, *μ_H_* and *μ_M_* are the error terms.

The main methodological obstacle is the endogeneity of migration. In line with the existing migration studies (e.g., [11,35,36]), we instrument the adult child migration with village-level indicators. The migration rate and the number of factories in a certain village are chosen as our instrumental variables. The rationale for this choice is discussed in Section 3.2.

### 4.2. Mechanism Design

In designing mechanisms for assessing the factors of the parental health, two groups of models are constructed. In the first group of models, the three inter-generational support variables are taken as dependent variables to test whether adult child migration exerts a significant impact on the inter-generational support obtained by the elderly parents. In the second group of models, three types of inter-generational support are included into the baseline model (Equation (1)) to test which types of inter-generational support exert a significant impact on parents’ health. We construct the models as follows:

(3) $${Metavariable}_{i}=\gamma_{0}+\gamma_{1}{Migration}_{i}+\gamma_{2}X_{i}+\gamma_{i}$$

(4) $${\mathrm{Health}}_{i}=\lambda_{0}+\lambda_{1}{Migration}_{i}+\lambda_{2}{Metavariable}_{i}+\lambda_{3}X_{H}+\lambda_{i}$$

where *Metavariable_i_* is the mediating variable, representing the remittance, farm work and emotional cohesion; *X_i_* is the vector of household and individual characteristics defined in Table 1.

## 5. Empirical Results

### 5.1. Main Results and Robustness Check

Table 2 (Column 1) shows the estimates for the migration decision model, i.e., the first-stage regression of the IV approach. The coefficients on the two instrumental variables (migration rate and number of factories) are both significant at the 1% level, and the F statistics are well above the rule-of-thumb threshold of 10 for weak instruments. The migration rate is highly significant and positively correlated with the likelihood of child migration as expected. In addition, the coefficient for *Factory* is significantly negative, implying that the increase in the activities of the industrial sector in hometown results in lower migration.

Table 2 (Columns 2–5) reports the empirical results of the second stage of the IV approach. More specifically, the estimate of coefficient for *Migration* indicates that decision to migrate is associated with lower SRH, physical (BMI and PADL), and mental health of the elderly parents in rural China.

Elderly women are more likely to report poor health than men. We also find that both higher education level of the elderly and higher household income in rural China are associated with better health of parents. It is likely that highly-educated and richer households can afford better nutrition, living conditions and health care [3]. The health of the spouse also affects the respondent’s health, especially when the spouse is in poor status of health, indicating that the ever-increasing care burden will mitigate the negative impact on respondent’s health. There are significantly positive impacts of children’s average education level on parental health. It is straightforward that adult children with higher-level education usually take care of their parents better. Moreover, we find that living with at least one adult child, son-in-law, or daughter-in-law in the same household is associated with better physical and mental health of parents. One of the reasons can be that if an adult child migrates to an urban area, the other young family members (i.e., other child, son-in-law, or daughter-in-law) take care of the left-behind parents instead.

To check robustness of the estimates, we also implemented the IV approach by using ordered logistic models at the second stage to examine the migration effect on parental health (the results are provided in Table A1). Our results show that both of the two models provide a similar qualitative picture, implying robustness of the results. Therefore, we mainly use the IV-2SLS model in the analysis.

### 5.2. Heterogeneous Effects

To comprehend the different effects of child migration on parents’ gender, years of education and living arrangement (Table 3), we first divide the sample into two groups by gender. The coefficient on *Migration* is higher in magnitude for the female group than that for the male group. Our results suggest female parents tend to suffer more from their child migration, which turn into poor SRH, and physical and mental health outcomes. It is possibly driven by the fact that, in the Chinese ‘patriarchy’ family system, most work such as doing house chores, and caring for the elderly and kids, has been highly feminized [37]. If the household has a migrant child, grandmothers will take on the bulk of the household chores and care for their grandchildren. This type of burden will damage their physical health. It is important to establish elderly support system to complement the traditional family care in rural China, although the traditional family-based support still plays an important role in rural China [3].

With respect to the level of education, adult child migration exerts a significantly negative impact on health of poorly-educated parents, but exerts no influence on health of highly-educated parents. This could be explained by assuming that the highly-educated parents often invest more in their health, e.g., pay more attention to balanced diets, take part in social security, and medical insurance schemes [38].

Living arrangement is an important factor affecting the available daily care of elderly parents. We have found that adult child migration exerts more significantly negative impacts on the health of parents who live with other adult children (also including son-in-law, and daughter-in-law) together, compared to those who live independently. One possible reason is children’s care influences parents’ self-assessment on their health and self-efficacy, and consequently causes parents’ excessive dependence on children [39], which conforms to the use and disuse theory.

Overall, our results indicate that child migration has higher adverse effects on parental health, typically for female cohorts, the less–educated groups, and the parents living with at least one adult in the same household.

### 5.3. Transmission Mechanism for Inter-Generational Support

The adverse effect of migrant children on parental health can be explained by considering the three types of inter-generational support, including money transfer, farm work, and emotional cohesion. In this section, we explore the transmission mechanism through which child migration affects their left-behind parents. Table 4 reports the estimates of the impact of child migration on inter-generational support.

We find that a migrant child can improve the financial conditions of left-behind parents. To some extent, migration can be used as an important means for relieving the poverty of families in the rural China [14]. Migrant children can strengthen their contacts with parents due to convenient modern telecommunication technologies. Still, child migration results in an increased parental workload on farm.

The model in Equation (4) is appended with each of the mediating variables to establish the second group of models shown in Table 5. Results in Column 2 of Table 5 show that the coefficient of money transferred by migrants to parents is not significantly positive. The income transfer to the elderly is a complicated phenomenon affected by children’s “willingness”, the elderly’s “needs”, and children’s “earning power” [14]. Due to limited human capital, low income and high living cost, the financial support transferred to parents left behind is limited and unstable, thus parents’ livelihood is not obviously improved. Additionally, elderly parents may spend most of the remittances on their family members (e.g., grandchildren), with little or no health-related expenses on themselves.

Besides the effect of remittances on health, another possible transmission mechanism is the change in time allocation. During the survey, the elderly were asked about the number of hours spent working on the family farm on a typical weekday. The results in Column 3 of Table 5 show that the left-behind parents spend more time on farm work, which poses a negative impact on their health. Due to the migration of adult children, the elderly allocate more time to work on farm suggesting that they substitute their labor, to a certain extent, for labor input previously provided by the migrant children.

Moreover, the health effect of child migration and time allocation is also embodied in the frequency of emotional cohesion. Column 4 of Table 5 shows that the inter-generational communication does not exert a significantly positive influence on left-behind parents’ SRH, although child migration significantly increases the communication between migrants and their left-behind parents (Table 4). One possible reason may be that although migrant children communicate with their parents more frequently, they may only pay more attention to their children, and care little about parents’ emotional solace. Admittedly, emotional exchanges between children and their parents will not cause parental loneliness and depression despite their long-distance separation.

These results deepen the understanding of the transmission mechanism of the negative effects. The major conclusion is that the negative influence of the burden of farm work on parental health outweighs the influence of economic support and emotional cohesion on parental health. Thus, the migration of adult children exerts a significantly negative impact on parental health (Column 1 of Table 5).

## 6. Discussion and Conclusions

Using the data from three latest issues of CHARLS for rural areas in China, this study employed the instrumental variable approach to investigate the impact of adult child migration on the health conditions of their parents left behind. We explored the relationship between child migration and the health of elderly parents through three types of inter-generational support (income transfer, farm work and emotional cohesion) for the first time. Our findings provide evidence that child migration significantly lowers parents’ SRH and physical health (i.e., BMI and PADL) and aggravates parents’ depression. From the point of view of inter-generational support, migration of the children induced the burden of farm work which, in turn, posed a significantly negative impact on the health of parents left behind. Moreover, the positive effect of economic support and emotional cohesion was weak and could not compensate for the adverse effect of the increased farm work. Overall, adult child migration exerts significantly negative impacts on parental health. Additionally, parents who are female, poorly-educated, and living with at least one adult child are the most vulnerable groups being in poor health outcomes.

The above-mentioned findings deepen the understanding of the role of traditional inter-generational support in the context of large-scale domestic emigration and population aging in China. Increasing burden of farm work is the major cause of affecting health of left-behind parents. Thus, the rural development policy in China should focus on reduction of farm work for the elderly. In the long-run, the young and middle-aged rural population will continue to leave their parents and opt for work in the cities of China. First of all, it is necessary to establish an effective healthcare insurance system, rural community medical services, and elderly people’s associations to guarantee the living conditions of left-behind seniors, especially, elderly women. Second, communities could establish the self-governing organizations among the elderly, which can increase their mutual assistance and reduce the resentment resulting from child migration. The burden of farm work of elderly parents left behind can be reduced through improved social service system, such as cooperatives or “cultivation assistance” groups that provide cultivation service for the left-behind elderly.

## Figures and Tables

**Table 1:** Average values for the variables used.

Variable	Definition	Whole Sample	Having Migrant Children	
			No	Yes
***Health measures***				
SRH	Self-reported health; 5 = excellent, 4 = very good, 3 = good, 2 = fair, 1 = poor	2.49	2.52	2.46 ***
BMI	Body mass index; weight (kg)/height ^2^ (m^2^)	23.30	23.38	23.19 ***
PADL	Physical activity of daily living; cumulative amount of activities accomplished by the respondent (7 items)	6.24	6.25	6.23 **
CES-D	Depression score with the sum of the 10 graded items (points)	18.60	18.38	18.83 ***
***Mediating variables***				
Remittance	Logged form of economic supports received from the non-cohabitant adult children (yuan/year)	4.85	3.99	5.93 ***
Farmwork	Doing farm work (hour/month)	54.36	49.70	60.23 ***
Emotional cohesion	The aggregate frequencies of emotional cohesion that adult children provide for parents	11.49	8.13	15.78 ***
***Migration measure***				
Migration	Having migrant children; 1 = yes, 0 = no	0.44	-	-
***Individual characteristics of the elderly***				
Age	Age (year)	60.27	59.83	60.83 ***
Gender	1 = male, 0 = female	0.46	0.45	0.47 **
Married	Having a spouse; 1 = yes, 0 = no	0.86	0.86	0.87 ***
Education	Years of education completed	4.44	4.45	4.43
Work	Working status in last year; 1 = yes, 0 = no	0.75	0.74	0.76 **
Diseases	Number of chronic diseases	1.29	1.24	1.35 ***
***Household characteristics***				
Income	Logged form of total household income (yuan/year)	6.72	6.93	6.46 ***
Number of children	Number of all children	3.88	3.75	4.05 ***
Average age of children	Years	27.99	27.55	28.56 ***
Average education of children	Years	7.21	7.08	7.39 ***
Living arrangement	At least one adult (son-in-law, daughter-in-law, or adult child) living in the same household; 1 = yes, 0 = no	0.45	0.50	0.37 ***
***Health of spouse***				
No spouse	Having no spouse; 1 = yes, 0 = no; reference group	0.19	0.20	0.17 ***
Healthy spouse	Having a healthy spouse; 1 = yes, 0 = no	0.38	0.38	0.38
Unhealthy spouse	Having an unhealthy spouse; 1 = yes, 0 = no	0.43	0.42	0.45 ***
***Instrumental variables***				
Migration rate	Migration rate of co-villagers (%)	0.31	0.27	0.37 ***
Factory	Number of factories in the resident village	5.33	6.40	3.97 ***
N	Number of observations	28,226	15,819	12,407

Data source: CHARLS (2011–2015). Note: ** and *** indicate significant differences in means for the two cohorts of parents at 5% and 1% level of statistical significance respectively.

**Table 2:** Baseline model of child migration and parental health.

Variable	Stage 1: Logistic Equation	Stage 2: Outcomes Equation			
	(1) Migration	(2) SRH	(3) BMI	(4) PADL	(5) CES−D
***Migration measure***					
Migration		−0.392 ***	−1.846 ***	−0.509 ***	1.346 **
		(0.105)	(0.350)	(0.110)	(0.636)
***Instrumental variables***					
Migration rate	0.475 ***				
	(0.047)				
Factory	−0.017 ***				
	(0.002)				
***Individual characteristics of the elderly***					
Age		0.001	−0.097 ***	−0.019 ***	−0.004
		(0.001)	(0.005)	(0.001)	(0.009)
Gender		0.076 ***	−0.830 ***	0.231 ***	−1.648 ***
		(0.015)	(0.051)	(0.015)	(0.097)
Married		−0.005	0.165 *	0.041	−1.827 ***
		(0.026)	(0.088)	(0.030)	(0.182)
Education		0.007 ***	0.041 ***	0.022 ***	−0.109 ***
		(0.002)	(0.007)	(0.002)	(0.013)
Work		0.229 ***	−0.397 ***	0.522 ***	−0.415 ***
		(0.017)	(0.060)	(0.021)	(0.119)
Diseases		−0.201 ***	0.277 ***	−0.169 ***	1.032 ***
		(0.005)	(0.018)	(0.006)	(0.036)
***Household characteristics***					
Income		0.007 ***	0.008	0.011 ***	−0.086 ***
		(0.002)	(0.007)	(0.002)	(0.014)
Number of children	0.117 ***	−0.003	0.020	0.010 *	0.026
	(0.009)	(0.006)	(0.019)	(0.006)	(0.036)
Average age of children	0.043 ***	−0.001	0.022 **	0.009 **	−0.0002
	(0.006)	(0.003)	(0.011)	(0.003)	(0.022)
Squared average age of children	0.0001	0.0001	0.0000	−0.0002 ***	−0.0005
	(0.006)	(0.0001)	(0.0002)	(0.0001)	(0.0003)
Average education of children	0.060 ***	0.017 ***	0.059 ***	0.024 ***	−0.147 ***
	(0.004)	(0.003)	(0.009)	(0.003)	(0.016)
Living arrangement		0.004	−0.047	0.035 **	−0.482 ***
		(0.017)	(0.057)	(0.017)	(0.109)
***Health of spouse***					
Healthy spouse		0.119 ***	0.114	0.014	−0.276 *
		(0.025)	(0.083)	(0.026)	(0.162)
Unhealthy spouse		−0.167 ***	−0.038	−0.105 ***	1.012 ***
		(0.024)	(0.082)	(0.026)	(0.163)

Notes: (1) *** *p* < 0.01; ** *p* < 0.05; * *p* < 0.10; (2) Values in parenthesis are robust standard errors; (3) The year and regional dummies are included in the models.

**Table 3:** Heterogeneity effects of child migration on parental health.

Groups	General Health	Physical Health		Mental Health
	(1) SAH	(2) BMI	(3) PADL	(4) CES−D10
1. Gender				
Male	−0.295	−1.605 ***	−0.452 ***	−0.170
Female	−0.447 ***	−2.023 ***	−0.587 ***	2.773 ***
2. Education level				
0−9 years	−0.427 ***	−1.886 ***	−0.559 ***	1.616 **
10 years and above	0.140	−1.880	−0.488	−1.086
3. Living arrangement				
Living with at least one adult	−0.857 ***	−4.583 ***	−0.691 ***	3.663 ***
Living independently	−0.196	−0.815 **	−0.422 ***	0.257

Notes: (1) *** *p* < 0.01; ** *p* < 0.05; (2) All models include a series of covariates (*X_H_*) shown in Table 1.

**Table 4:** The effect of child migration on inter-generational supports.

Variable	Remittance	Farmwork	Emotional Cohesion
Migration	4.834 ***	61.472 ***	26.995 ***
	(0.286)	(5.819)	(0.893)
Age	0.048 ***	−0.651 ***	0.162 ***
	(0.004)	(0.089)	(0.013)
Diseases	0.069 ***	−2.285 ***	0.211 ***
	(0.172)	(0.376)	(0.050)
Average age of children	0.132 ***	1.287 ***	0.172 ***
	(0.011)	(0.222)	(0.031)
Squared average age of children	−0.002 ***	−0.028 ***	−0.002 ***
	(0.0002)	(0.004)	(0.001)
Healthy spouse	0.100	12.458 ***	1.410 ***
	(0.067)	(1.459)	(0.201)
Unhealthy spouse	0.239 ***	17.739 ***	1.736 ***
	(0.064)	(1.399)	(0.197)

Notes: (1) *** *p* < 0.01; (2) The year and regional dummies are included in the models.

**Table 5:** The health effect of child migration and inter-generational support (SRH).

Variable	(1)	(2)	(3)	(4)
***Migration measure***				
Migration	−0.392 ***	−0.400 ***	−0.361 ***	−0.407 ***
	(0.105)	(0.105)	(0.105)	(0.105)
***Mediating variables***				
Remittances		0.001		
		(0.002)		
Farmwork			−0.0002 **	
			(0.0001)	
Emotional cohesion				0.001
				(0.001)
***Individual characteristics of the elderly***				
Age	0.001	0.0005	0.001	0.001
	(0.001)	(0.001)	(0.001)	(0.001)
Gender	0.076 ***	0.076 ***	0.076 ***	0.076 ***
	(0.015)	(0.015)	(0.015)	(0.015)
Married	−0.005	−0.005	−0.004	−0.007
	(0.026)	(0.026)	(0.026)	(0.026)
Education	0.007 ***	0.007 ***	0.007 ***	0.007 ***
	(0.002)	(0.002)	(0.002)	(0.002)
Work	0.229 ***	0.228 ***	0.248 ***	0.228 ***
	(0.017)	(0.017)	(0.019)	(0.017)
Diseases	−0.201 ***	−0.201 ***	−0.201 ***	−0.201 ***
	(0.005)	(0.005)	(0.005)	(0.005)
***Household characteristics***				
Income	0.007 ***	0.007 ***	0.007 ***	0.008 ***
	(0.002)	(0.002)	(0.002)	(0.002)
Number of children	−0.003	−0.003	−0.004	−0.005
	(0.006)	(0.006)	(0.006)	(0.006)
Average age of children	−0.001	−0.002	−0.002	−0.002
	(0.003)	(0.003)	(0.003)	(0.003)
Squared average age of children		0.0001	0.0001	0.0001
		(0.0001)	(0.0001)	(0.0001)
Average education of children	0.017 ***	0.017 ***	0.017 ***	0.017 ***
	(0.003)	(0.003)	(0.003)	(0.003)
Living arrangement	0.004	0.005	0.004	0.011
	(0.017)	(0.017)	(0.017)	(0.018)
Healthy spouse	0.119 ***	0.120 ***	0.121 ***	0.120 ***
	(0.025)	(0.025)	(0.025)	(0.025)
Unhealthy spouse	−0.167 ***	−0.167 ***	−0.164 ***	−0.167 ***
	(0.024)	(0.024)	(0.024)	(0.024)

Notes: (1) *** *p* < 0.01; ** *p* < 0.05; (2) All models include a series of covariates (*X*) shown in Table 1; (3) Only parents’ SRH is gauged in models (2)–(4) as an example; (4) The result in Column 2 of Table 2 is also shown in Column 1 of Table 5 for comparison.

## Appendix A

In the CHARLS, a respondent’s SRH forms a continuum from excellent to poor health, gauged by five options (1 = excellent, 2 = very good, 3 = good, 4 = fair, and 5 = poor). We reverse the scale to be an increasing ordinal so that 1 represents “poor”, while 5 for “excellent”. As a subjective measure, SRH may be more susceptible to misreporting due to the health-related knowledge, including personal values, beliefs, and information (Huang et al., 2016). Nevertheless, several studies demonstrated that subjective measures of health are good at predicting objective health conditions [40], including morbidity and mortality.

For the physical health outcome, two variables are used in the analysis. One is BMI computed as the ratio of weight (kg) to the square of height (m^2^) in a standard defined by the World Health Organization. BMI is included since it reflects some direct health practices, such as dietary and exercise habits. Thus, BMI has been widely used to gauge the fitness of adults. The other variable we construct is PADL defined as the cumulative amount of activities that can be accomplished by the respondent. This indicator is used to measure physical fitness, mainly including seven items such as weight bearing, crouching, and jogging, etc.

Depression is a recognized mental health problem that adversely impact upon the individual’s ability to function and daily life. A 10-item Center for the Epidemiological Studies of Depression (CES-D10) is a widely used self-report measure of depression symptomatology during the past week [41]. Four options is included and can be graded according to a Likert scale: “rarely or none of the time, <1 day (1 point)”, “some or a little of the time, 1–2 days (2 points)”, “occasionally or a moderate amount of time, 3–4 days (3 points)”, and “all the time, 5–7 days (4 points)”. Final score is the sum of the 10 graded items, and ranges from 10 to 40, with higher scores indicating a greater frequency of depressed mood [42].

For the robust results, we convert the health quality variables (i.e., BMI and CES-D) into dichotomic outcomes, i.e., 18 < BMI < 25 is taken as health, and it is valued as 1, or valued as 0; The “poor mental health” (CES-D score > 20) is equal to one if the respondent reports having felt depressed, lonely, or sad for the majority of the time in last week, otherwise equal to zero. The definitions and values of SRH and PADL, as multivariate discrete variables, remain unchanged in the robustness of estimation.

## Appendix B

**Table A1:** Robustness Estimation of child migration and parental health: IV-(ordered) logistic model.

Variable	(1) SRH	(2) BMI	(3) PADL	(4) CES−D
***Migration measure***				
Migration	−0.739 ***	−0.797 *	−1.161 ***	0.476 **
	(0.198)	(0.420)	(0.228)	(0.243)
***Individual characteristics of the elderly***				
Age	0.002	−0.090 ***	−0.043 ***	0.010 ***
	(0.003)	(0.006)	(0.003)	(0.003)
Gender	0.134 ***	0.083	0.568 ***	−0.577 ***
	(0.029)	(0.074)	(0.033)	(0.035)
Married	−0.020	−0.197 *	0.036	−0.429 ***
	(0.049)	(0.117)	(0.054)	(0.058)
Education	0.016 ***	−0.004	0.058 ***	−0.042 ***
	(0.004)	(0.011)	(0.005)	(0.005)
work	0.461 ***	0.081	0.858 ***	−0.162 ***
	(0.035)	(0.080)	(0.035)	(0.038)
Diseases	−0.398 ***	0.043 *	−0.340 ***	0.277 ***
	(0.011)	(0.024)	(0.011)	(0.012)
***Household characteristics***				
Income	0.013 ***	−0.017 *	0.022 ***	−0.029 ***
	(0.004)	(0.010)	(0.004)	(0.005)
Number of children	−0.007	0.021	0.038 ***	0.003
	(0.011)	(0.024)	(0.012)	(0.013)
Average age of children	−0.002	−0.015	0.001	−0.014 *
	(0.006)	(0.015)	(0.007)	(0.007)
Squared average age of children	0.0001	0.0005 **	−0.0000	0.0001
	(0.0001)	(0.0002)	(0.0001)	(0.0001)
Average education of children	0.033 ***	0.047 ***	0.053 ***	−0.046 ***
	(0.005)	(0.013)	(0.005)	(0.006)
Living arrangement	0.021	−0.066	0.075 **	−0.093 **
	(0.033)	(0.080)	(0.036)	(0.039)
Health of spouse				
Healthy spouse	0.247 ***	0.082	0.045	−0.105 *
	(0.048)	(0.121)	(0.053)	(0.056)
Unhealthy spouse	−0.300 ***	0.006	−0.231 ***	0.258 ***
	(0.046)	(0.114)	(0.051)	(0.054)

Notes: (1) *** *p* < 0.01; ** *p* < 0.05; * *p* < 0.10; (2) Values in parenthesis are robust standard errors; (3) The year and regional variables are also controlled and coded as dummy variables in models.
